# Social media addiction and psychological outcomes: the mediating roles of affect, authenticity, and self-image

**DOI:** 10.3389/fpsyg.2026.1837689

**Published:** 2026-05-18

**Authors:** Andra Seceleanu, Irina Sunda, Bianca Ioana Garabet

**Affiliations:** Department of Psychology, Faculty of Psychology, Behavioral and Legal Sciences, Andrei Șaguna University, Constanța, Romania

**Keywords:** cognitive autonomy, emotional regulation, flourishing, identity processes, neurorights, self-concept, social media addiction

## Abstract

The expansion of social media as a dominant infrastructure of social interaction has raised important questions regarding its impact on cognitive, emotional, and identity-related processes. While prior research has predominantly relied on exposure-based models, increasing attention is directed toward the ways through which digitally mediated environments influence psychological functioning. At the same time, emerging interdisciplinary frameworks in neurolaw and neurorights highlight concerns related to mental integrity, cognitive liberty, and mental privacy, emphasizing the need to understand how algorithmically structured environments interact with internal psychological processes. The present study examines the psychological ways linking social media addiction to academic performance and psychological flourishing, while providing an empirically grounded perspective relevant to digital governance. A cross-sectional sample of 940 participants (aged 18–72 years; M = 31.00, SD = 11.35) completed validated measures of social media addiction, affectivity, authenticity, self-image, academic performance, and flourishing. A path analysis with observed composite scores was used to test a mediation model. Results indicate that social media addiction, distinct from general usage, was associated with higher negative affect, lower positive affect, and lower authenticity. These associations were statistically mediated through self-image in a cross-sectional covariance model, with self-image emerging as the strongest indirect pathway linking compulsive digital engagement to lower academic performance and reduced psychological flourishing. Among the mediating variables, authenticity represented the strongest indirect association, followed by affective processes, whereas non-compulsive use showed no significant associations. These findings support a mechanism-based model in which the impact of social media operates through affective dysregulation and identity-related processes rather than direct exposure. From an interpretative perspective, the results are conceptually consistent with concerns raised in neurorights discourse regarding cognitive autonomy and mental integrity. These findings should be understood as psychological correlates that may be relevant to such debates; they do not constitute direct evidence of rights violations or neuropsychological harm, and no neurocognitive variables were assessed in this study. Overall, the study contributes to a more precise understanding of digital behavior by integrating psychological statistical mediators with broader frameworks of digital governance and provides a foundation for interventions targeting internal processes while preserving fundamental freedoms such as expression and privacy.

## 1 Introduction

Rapid advancements in digital technologies have prompted growing interdisciplinary interest in their effects on psychological functioning and human rights. As an emerging field, neurolaw examines how digital and neurotechnological systems challenge ethical and legal frameworks, particularly with respect to cognitive autonomy, mental integrity, and personal identity (Neuwirth, 2022; Ienca, 2023). Algorithmic systems embedded in social media platforms rely on persuasive architectures, including dark patterns, hypernudging, and personalization, that exploit cognitive vulnerabilities and may reduce users’ capacity for informed, autonomous decision-making (Carlessi et al., 2023; Pedrosa, 2024). These concerns have motivated the development of neurorights frameworks, which propose legal protections for the mental domain (Cornejo, 2024; Farinella and Gulyaeva, 2024). However, these frameworks remain largely normative; their empirical grounding in observable psychological processes is limited, and this gap represents an important motivation for the present study. It is important to note that the present study does not directly measure or test neurorights constructs. Rather, key psychological variables, authenticity and self-image, are treated as functional indicators of processes that may be conceptually relevant to debates about cognitive autonomy and mental integrity. This interpretive bridge is supported by work demonstrating that identity and agency are dissociable dimensions of selfhood that may be differentially affected by technological environments (Di Plinio et al., 2020), and by scholarship linking algorithmically mediated influence to threats to psychological autonomy (Di Plinio et al., 2020; Ienca, 2021). Authenticity, operationalized as the congruence between internal states and external self-expression, serves as a proxy for identity-related autonomy. Self-image reflects the degree to which externally mediated influences have been internalized into the self-concept. These variables are psychological correlates potentially relevant to neurorights debates, not empirical proxies for rights violations.

These theoretical concerns are reinforced by the rapid development of neurotechnologies and AI systems capable of accessing, modeling, and influencing cognitive processes. BCIs enable direct interaction between brain activity and external devices, raising questions about neural data governance and cognitive surveillance (Trabucco, 2023; Neuwirth, 2022). At the same time, AI-driven systems can infer emotional states and behavioral tendencies from digital traces, extending neurorights concerns to indirect forms of “mind inference” (Muñoz et al., 2023; Brown, 2024). In more advanced scenarios, such technologies may alter agency and self-perception, potentially leading to unauthorized forms of cognitive influence (Andorno and Lavazza, 2023; Chang, 2025; Goering et al., 2021; Istace, 2022; Pugh et al., 2018; Botes, 2022).

These dynamics are particularly evident in social media platforms, where interface design and algorithmic systems are optimized to capture and sustain user attention. Dark patterns and persuasive architectures exploit cognitive biases and social pressures, increasing the likelihood of unintended behaviors and reducing users’ capacity for informed decision-making (Carlessi et al., 2023; Singh et al., 2023; Roșca, 2024; Shikh Alghanameh and Aljazaeri, 2024). Empirical evidence suggests that such designs increase compliance with unwanted actions and may operate through conditional obedience, de-individuation, and learned helplessness (Luguri and Strahilevitz, 2019; Dresp-Langley, 2024). More broadly, these processes are embedded within the logic of the attention economy, in which user engagement is transformed into economic value and behavioral data become a resource for prediction and influence (Giraldo-Luque and Fernández-Rovira, 2021; Bahytjanuly, 2025; Kasper et al., 2023).

Although these developments are often discussed in legal and policy terms, their practical relevance depends on whether they can be translated into observable psychological processes. Social media platforms provide a particularly important context for such analysis, as they combine large-scale data extraction with algorithmic personalization capable of influencing both behavior and internal mental states. From a psychological perspective, these platforms function as behaviorally engineered environments in which emotional reactions, attentional patterns, and self-representations become both inputs and targets of algorithmic systems.

Psychological research on problematic social media use offers an important empirical lens for examining these dynamics. Previous studies have linked problematic or addictive engagement with negative affect, lower well-being, maladaptive social comparison, and reduced self-regulatory capacity. These findings suggest that the impact of digital environments is not determined by usage alone, but by how platform structures interact with affective and identity-related processes.

The present study builds on this perspective by integrating neurorights theory with empirical psychological modeling. Rather than treating neurorights as directly measurable constructs, it interprets key psychological variables as functional indicators of processes relevant to cognitive autonomy and mental integrity. Within this framework, authenticity can be understood as a proxy for identity-related autonomy, reflecting the degree of congruence between internal states and external self-expression in digitally mediated environments. Affectivity, particularly elevated negative affect and reduced positive affect, can be interpreted as an indicator of cognitive and emotional vulnerability, potentially increasing susceptibility to persuasive technologies. Self-image represents the level at which externally mediated influences are internalized into the self-concept, and may therefore reflect deeper impacts on mental integrity and psychological continuity.

By establishing this conceptual bridge, the study shifts the focus from exposure-based models toward a mechanism-based framework that examines how digital environments affect psychological functioning through affective and identity-related pathways. Accordingly, the present research investigates the roles of affect, authenticity, and self-image in explaining how social media addiction relates to academic performance and psychological flourishing.

The use of perceived academic performance as an outcome in a general adult sample requires justification. While this measure is most commonly employed in student populations, it was included here as an indicator of functional cognitive productivity and self-assessed learning efficacy, constructs that retain relevance across educational and professional contexts (York et al., 2015). The broad age range of the present sample (18–72 years) reflects the diversity of social media users in real-world contexts, and the inclusion of a performance-related outcome allows examination of whether social media addiction has functional consequences beyond subjective well-being. Nonetheless, readers should interpret findings related to academic performance cautiously in relation to participants who are not currently enrolled in formal education.

Despite the rapid expansion of research on social media and well-being, the empirical literature remains inconsistent. Large-scale and meta-analytic studies have reported weak or negligible associations between social media use and well-being (Orben and Przybylski, 2019; Huang, 2017), while others document meaningful negative effects, particularly in the context of compulsive or addictive engagement (Andreassen et al., 2016b; Marino et al., 2018). These inconsistencies suggest that exposure-based models, focusing on time spent online or frequency of use, are insufficient, and that the psychological processes through which digital engagement affects outcomes remain poorly understood. A further gap concerns the specific mediating variables examined here: affect, authenticity, and self-image have rarely been studied in combination, and their roles as mediators linking social media addiction to performance and well-being outcomes have not been systematically tested. The present study addresses both of these gaps by proposing and testing a mechanism-based model that moves beyond exposure to examine the internal processes through which addictive engagement affects adaptive outcomes.

Addressing this gap requires an integrative approach that links abstract normative frameworks with measurable psychological statistical mediators. Specifically, there is a need to identify whether and how affective processes and identity-related constructs function as pathways through which social media engagement impacts well-being and performance. By combining insights from digital psychology, identity research, and neurorights theory, the present study moves beyond descriptive associations and proposes a mechanism-based model of digital influence.

## 2 Conceptual model and hypotheses

Accordingly, the present study adopts a mechanism-based perspective to examine how social media addiction may influence adaptive outcomes through internal psychological processes. Building on neurorights-informed and neurocognitive frameworks, the study focuses on affectivity, authenticity, and self-image as key mechanisms through which digitally mediated environments may shape emotional regulation, identity construction, and self-evaluation.

Specifically, the research tests a mediation model in which social media addiction is associated with increased negative affect, reduced positive affect, and lower authenticity, which in turn influence self-image. Self-image is conceptualized as a central integrative construct linking affective and identity-related processes to academic performance and psychological flourishing. This approach enables a more precise understanding of how digital engagement becomes maladaptive, not through exposure per se, but through its psychological internalization.

Based on this framework, the following hypotheses were formulated:

*H1*: Social media addiction is negatively associated with self-image, such that higher levels of addictive engagement are linked to lower self-evaluation.

*H2*: Affectivity mediates the relationship between social media addiction and self-image, such that negative affect amplifies, whereas positive affect attenuates this relationship.

*H3*: Authenticity mediates the relationship between social media addiction and self-image, such that higher authenticity is associated with a more positive self-image.

*H4*: Self-image mediates the relationship between social media addiction and academic performance.

*H5*: Self-image mediates the relationship between social media addiction and psychological flourishing.

## 3 Methods

### 3.1 Study design, setting, and ethics

The present study employed a cross-sectional, correlational design using an online questionnaire. The survey link was distributed via social media and email to facilitate anonymous participation among adults.

On the first page of the questionnaire, participants were informed about the aims of the study, the voluntary nature of participation, the protection of confidentiality and anonymity, and their right to discontinue participation at any time by closing the browser window, without any identifying information being collected. Data collection took place in a remote, self-paced context, allowing respondents to complete the instruments in an environment of their choice (Booker et al., 2021).

The study protocol was approved by the Ethics and Integrity Commission of Andrei Șaguna University, Constanța, Romania, in February 2025 (Approval No. 26/2025).

### 3.2 Participants and procedure

A total of 941 individuals accessed the survey. After excluding one case with substantial missing data, the final analytic sample consisted of 940 participants. Participants ranged in age from 18 to 72 years (M = 31.00, SD = 11.35). The age distribution reflected a broad profile, spanning both younger participants typically associated with academic contexts and older adults in later stages of professional development. The sample remained concentrated primarily in younger age groups, with 44.3% aged 18–25 years, 43.1% aged 26–45 years, 12.1% aged 46–60 years, and 0.5% aged over 60.

The sample included 694 women, 235 men, and 11 participants who identified as another gender or preferred not to disclose their gender. Participants completed a battery of self-report instruments administered online, requiring approximately 15–20 min.

The respondents ranged in age from 18 to 72 years, reflecting a broad demographic profile that included both younger individuals typically associated with academic settings and adults in later stages of their professional careers. The age distribution was centered primarily on younger participants between 18 and 30 years, which is consistent with the contemporary university environment. The most represented ages were 20 years (10.3%), 19 years (9.0%), 21 years (6.6%), and 22 years (6.1%), with frequencies gradually decreasing across older age groups. This demographic structure is appropriate for the aims of the study, given that younger adults represent the segment most intensively exposed to the digital ecosystem.

The questionnaire was distributed predominantly through a snowball sampling procedure using institutional and academic networks from Andrei Șaguna University, Politehnica University, the University of Craiova, UMF Craiova (including individuals enrolled in continuing professional education programs), Dimitrie Cantemir University of Târgu Mureș, SNSPA, and Ovidius University. Participants were recruited from undergraduate, master’s, and doctoral programs, contributing to a sample with meaningful diversity in educational level and academic maturity.

Responses were automatically recorded in a password-protected database. At the end of the questionnaire, participants received a brief debriefing regarding the study’s aims. No financial or material compensation was provided.

Data quality was assessed by inspecting response distributions and completion patterns, including response time, to identify implausibly rapid submissions. No additional cases were excluded based on these criteria.

### 3.3 Measures

To test the proposed model, a set of validated and study-specific instruments was employed to capture digital behavior, affective processes, identity-related mechanisms, and adaptive outcomes. All measures demonstrated acceptable to excellent internal consistency in the analyzed sample. The instruments were selected to ensure conceptual alignment with the theoretical framework and adequate coverage of the latent constructs included in path analysis.

#### 3.3.1 Problematic social media use (SMU)

Problematic social media use (SMU) was assessed using a scale developed within the present study to capture patterns of potentially maladaptive engagement across major social networking platforms. The instrument included items reflecting frequency of use, daily time spent online, and the primary purpose of platform use. Participants indicated whether their engagement was oriented toward information and news consumption, social interaction, entertainment, or professional activities, allowing a distinction between functional and reward-driven patterns of use.

Responses were recorded on a five-point Likert scale ranging from 1 (“never”) to 5 (“always”). For items in which higher scores reflected lower levels of usage, a linear transformation (12 - score) was applied to reverse their direction, so that higher values consistently indicated higher levels of use across all items. For a five-point Likert scale with a minimum score of 1 and maximum of 5, this transformation yields a range of 7 to 11, preserving the interval structure while reversing item polarity. This transformation was applied solely for interpretive consistency and did not affect the underlying statistical relationships among variables.

Internal consistency was acceptable to good across platform-specific subscales, with Cronbach’s alpha coefficients ranging from 0.74 to 0.88 (Facebook: 0.74–0.77; Instagram: 0.79–0.83; Snapchat: 0.75–0.76; TikTok: 0.86–0.88). In addition, the hierarchical omega coefficient indicated adequate support for computing a composite score (*ω*_h_ = 0.61; *ω*_total = 0.91), suggesting that the scale can be interpreted as a cumulative index despite moderate hierarchical saturation. To examine the factorial validity of this scale, a confirmatory factor analysis was conducted on the four platform-level composite scores (Facebook, Instagram, TikTok, Snapchat) as indicators of a single latent SMU factor. The model was estimated using MLR estimation with FIML for missing data (*N* = 941). Global fit indices were excellent: *χ*^2^(2) = 2.48, *p* = 0.290, CFI = 0.999, TLI = 0.997, RMSEA = 0.018 (90% CI [0.000, 0.076]), SRMR = 0.011. However, the parameter estimates revealed substantial interpretive problems. The factor loading for Facebook was near zero (*λ* = 0.034), while the remaining three platform indicators produced large negative loadings (Instagram: *λ* = −0.646; TikTok: *λ* = −0.776; Snapchat: *λ* = −0.660), and the latent factor variance was negligible and non-significant (variance = 0.004, *p* = 0.680). This pattern indicates that the four platform composites do not cohere as indicators of a single underlying construct, and the excellent global fit is likely an artifact of the model’s minimal degrees of freedom (df = 2) rather than genuine construct validity. These results indicate limited construct validity for the composite index as a unidimensional measure. Given that this is a newly developed scale, replication and validation in independent samples remain necessary. Accordingly, findings involving the SMU predictor should be interpreted as exploratory, and the null results for this variable should not be taken as definitive evidence against exposure-based accounts of social media harm without further validation of the measure itself.

The composite index was retained as an observed predictor in the structural model. Conceptually, this measure captures general intensity and patterns of use rather than compulsive or addiction-like engagement. It is therefore more accurately described as a usage index than a measure of problematic use in a clinical or behavioral-addiction sense. This distinction is important when interpreting null findings for this predictor: the absence of significant associations may partly reflect how the construct is defined rather than constituting strong evidence against exposure-based accounts of social media harm.

#### 3.3.2 Social media addiction (BSMAS)

Social media addiction was assessed using the Bergen Social Media Addiction Scale (BSMAS; Andreassen et al., 2016a), in its 6-item version culturally adapted for the Romanian population. The scale evaluates addictive engagement with social media through six items reflecting the core components of behavioral addiction: salience, mood modification, tolerance, withdrawal, conflict, and relapse.

Participants rated each item on a five-point Likert scale ranging from 1 (“very rarely”) to 5 (“very often”). In the present study, the scale demonstrated excellent internal consistency (Cronbach’s *α* = 0.89), indicating high reliability and item coherence.

BSMAS is widely recognized as a standard instrument for assessing social media addiction and shows strong convergent validity with other behavioral addiction measures. Within the present model, it represents the core indicator of compulsive and dysregulated engagement and is conceptually distinct from general patterns of use captured by the SMU scale.

#### 3.3.3 Affectivity (PANAS)

Affectivity was measured using the Positive and Negative Affect Schedule (PANAS; Watson et al., 1988), a widely used instrument comprising 20 items grouped into two dimensions: positive affect and negative affect.

Participants rated the extent to which they experienced each emotional state on a five-point Likert scale. Positive affect reflects energy, enthusiasm, and engagement, whereas negative affect reflects distress, anxiety, irritability, and emotional discomfort.

In the present sample, internal consistency was excellent for both subscales, with Cronbach’s *α* = 0.90 for positive affect and *α* = 0.91 for negative affect. These dimensions were modeled as mediators linking social media addiction to identity-related processes.

#### 3.3.4 Authenticity

Authenticity was assessed using the Authenticity Scale (Wood et al., 2008), which conceptualizes authenticity as a multidimensional construct comprising awareness, unbiased processing, and behavior and relational orientation.

Participants responded on a five-point Likert scale. Following inspection of distributional properties and internal consistency, one item from the Awareness subscale was removed in order to optimize reliability.

In the final version, subscale reliabilities were as follows: *α* = 0.69 for Awareness, *α* = 0.67 for Unbiased Processing, and *α* = 0.75 for Behavior and Relational Orientation. It should be noted that two of the three subscales fall below the conventional *α* = 0.70 threshold. Combined with the *post hoc* removal of one item, these values indicate that the authenticity measure is psychometrically fragile in the present sample. This limitation is particularly important to keep in mind when interpreting the structural findings, given that authenticity subsequently emerges as the strongest indirect pathway in the model. Conclusions regarding authenticity should therefore be treated with appropriate caution. To clarify, authenticity in this study was operationalized according to the theoretical framework of Wood et al. (2008), which defines authenticity as a multidimensional construct made up of three components: Awareness (awareness of one’s own motives, feelings, and desires), Unbiased Processing (the ability to process self-relevant information in a balanced and objective way), and Behavior and Relational Orientation (acting in accordance with one’s values in relationships). These three subscales were used as indicators of a latent authenticity construct within the SEM framework. This conceptualization aligns with literature that positions authenticity as the degree of congruence between internal experiences and external self-expression, and is supported by evidence linking higher authenticity to greater psychological well-being (Reinecke and Trepte, 2014; Wood et al., 2008).

#### 3.3.5 Self-image (PAS)

The PAS (Constantin, 2018) is a broad 84-item instrument originally validated on a Romanian adult sample and designed to assess multiple dimensions of the self. It comprises four major dimensions, each measured through 21 items grouped into three factors. Responses are recorded on a six-point Likert scale ranging from 0 (“Never true”) to 5 (“Always true”). Scores are interpreted relative to age- and gender-specific norms, allowing the identification of a psychological profile of self-perceptions and attitudes across four domains: self-image (self-esteem, self-efficacy, achievement), social desirability (self-deception, self-presentation, exaggeration), self-devaluation (gratitude, regret, self-deprecation), and moral integrity (sincerity, honesty, morality).

For the present study, 21 items from the full scale were retained to assess three core self-image subscales: self-esteem (7 items), self-efficacy (7 items), and self-achievement (7 items). The reliabilities of these three subscales were high (*α* ranging from 0.88 to 0.91), indicating very good accuracy in the measurement of these identity-related constructs. The instrument is frequently used in research on personal development, academic adaptation, and complex psychological assessment. In the structural model, these three components were specified as indicators of a latent self-image construct, conceptualized as a central integrative dimension reflecting evaluative, motivational, and achievement-related aspects of the self.

#### 3.3.6 Academic performance (APS)

Perceived academic performance was measured using the Academic Performance Scale (APS; York et al., 2015), which assesses perceived academic progress, learning-related task efficiency, and overall academic functioning.

The instrument includes six items rated on a five-point Likert scale. Higher scores indicate higher perceived academic competence. In the present study, the scale demonstrated very good internal consistency (Cronbach’s *α* = 0.88).

Academic performance was modeled as an outcome variable reflecting functional adaptation in the educational domain.

Given that the sample included adults ranging from 18 to 72 years of age, not all of whom were necessarily current students, a clarification regarding the applicability of this measure is warranted. Perceived academic performance was interpreted broadly as reflecting functional learning efficacy and self-assessed cognitive productivity, which may be relevant across educational and professional contexts. All other participants were excluded from models involving this outcome variable.

#### 3.3.7 Psychological flourishing

Psychological well-being was assessed using the Flourishing Scale (Diener et al., 2010), an eight-item instrument capturing key aspects of positive functioning, including meaning in life, social relationships, competence, and contribution to others.

Responses were recorded on a seven-point Likert scale. Internal consistency was excellent in the present sample (Cronbach’s *α* = 0.90).

Flourishing was modeled as a global indicator of psychological well-being and adaptive functioning.

Taken together, the instruments provided comprehensive coverage of the conceptual domains included in the theoretical model. Digital behavior was assessed using SMU and BSMAS; affective processes using PANAS; identity-related approaches using authenticity and self-image measures; and adaptive outcomes using academic performance and flourishing. This integrated measurement framework enabled testing a multilevel mediation model linking digital engagement to psychological and academic functioning.

### 3.4 Statistical analyses

All analyses were conducted in R (Version 4.5.1; R Core Team, 2025), using several packages for data processing and statistical analysis, including dplyr (Wickham et al., 2023), googledrive (D’Agostino McGowan and Bryan, 2025), googlesheets4 (Bryan, 2025), haven (Wickham et al., 2025), Hmisc (Harrell, 2025), kableExtra (Zhu, 2024), mvtnorm (Genz and Bretz, 2009), papaja (Aust and Barth, 2024), psych (Revelle, 2025), rstatix (Kassambara, 2025), sasLM (Bae, 2025), and tinylabels (Barth, 2025).

Descriptive statistics, internal consistency coefficients, and bivariate associations were computed for all study variables. Because the assumption of univariate normality was not met for any of the analyzed variables (all Shapiro–Wilk tests *p* < 0.001), and multivariate normality was also violated according to Mardia’s indicators, subsequent analyses relied on robust statistical procedures. Specifically, multivariate assessment indicated Mahalanobis distances ranging from 1.18 to 7.69, significant positive multivariate skewness (*Mardia* = 14.03, *Skewness* = 2200.23, *p* < 0.001), and leptokurtic multivariate distribution (*Mardia* = 290.18, *Kurtosis* = 23.90, *p* < 0.001).

Given these distributional properties, associations among variables were examined using Spearman correlations. The proposed model was estimated using robust path analysis using observed composite scores implemented in the lavaan package (Rosseel, 2012) with maximum likelihood estimation with robust standard errors and a Satorra–Bentler scaled test statistic (MLR) to account for non-normality. Ordinal items were treated as continuous indicators. Ordinal items were treated as continuous indicators. Missing data were handled using listwise deletion. All reported path coefficients are standardized. Indirect effects were tested using bootstrapped confidence intervals. No *post hoc* residual covariances were introduced. Direct, indirect, and total effects are reported with 95% confidence intervals.

It is acknowledged that the authenticity measurement model demonstrated poor fit (CFI = 0.755, TLI = 0.671, RMSEA = 0.170, SRMR = 0.110). Given that authenticity is the strongest mediator in the structural model, this poses an interpretive challenge: structural parameter estimates involving a poorly fitting latent construct may not accurately reflect the underlying construct relationships. To address this, the authenticity-specific indirect effects are reported separately and interpreted with explicit caution given the measurement model limitations. Conclusions regarding the role of authenticity in the model are therefore treated as preliminary and in need of replication with a better-fitting operationalization.

## 4 Results

### 4.1 Descriptive statistics and correlations

Descriptive statistics and correlation coefficients are presented in Tables 1, 2. Mean scores indicated moderate levels of social media addiction (*M* = 12.55, *SD* = 5.19), relatively high positive affect (*M* = 32.62, *SD* = 8.34), and moderate negative affect (*M* = 21.24, *SD* = 8.48). Self-related variables and adaptive outcomes also showed relatively elevated mean values, including self-esteem (*M* = 27.25, *SD* = 5.49), self-efficacy (*M* = 27.69, *SD* = 4.77), academic performance (*M* = 22.45, *SD* = 5.01), and flourishing (*M* = 45.70, *SD* = 8.66). All scales demonstrated satisfactory to excellent internal consistency, with Cronbach’s alpha coefficients ranging from 0.67 to 0.91.

**Table 1:** Distribution of participants by gender.

Gender	Frequency (*N*)	Percentage (%)	Valid percentage (%)	Cumulative (%)
Women	694	73.8	73.8	73.8
Men	235	25.0	25.0	98.8
Other gender/prefer not to say	11	1.2	1.2	100.0
Total valid	**940**	**100.0**	**100.0**	—

Bold values indicates total valid counts and percentages.

**Table 2:** Distribution of participants by age group.

Age group	Frequency (*n*)	Valid percentage (%)
18–25 years	430	45.7
26–45 years	405	43.1
46–60 years	114	12.1
60 + years	5	0.5
Total	**940**	**100.0**

Bold values indicates total valid counts and percentages.

Spearman correlation analyses revealed a coherent pattern of associations among the main variables. Social media addiction was positively associated with negative affect (*r* = 0.34, *p* < 0.001) and negatively associated with positive affect (*r* = −0.16, *p* < 0.001), awareness (*r* = −0.23, *p* < 0.001), behavior and relational orientation (*r* = −0.40, *p* < 0.001), self-esteem (*r* = −0.34, *p* < 0.001), self-efficacy (*r* = −0.30, *p* < 0.001), self-achievement (*r* = −0.29, *p* < 0.001), academic performance (*r* = −0.18, *p* < 0.001), and flourishing (*r* = −0.29, *p* < 0.001). Positive affect was positively associated with self-related variables and adaptive outcomes, whereas negative affect showed the opposite pattern. Overall, the correlation matrix was consistent with a mediated model in which the effects of social media addiction on performance and well-being are transmitted through affective and identity-related processes.

The measurement models showed mixed fit across constructs. The BSMAS model demonstrated acceptable fit, *χ*^2^(9) = 92.13, *p* < 0.001, CFI = 0.925, TLI = 0.876, RMSEA = 0.120 (90% CI [0.099, 0.143]), SRMR = 0.042. The authenticity model showed poor fit, *χ*^2^(41) = 928.07, *p* < 0.001, CFI = 0.755, TLI = 0.671, RMSEA = 0.170 (90% CI [0.161, 0.180]), SRMR = 0.110. The self-image model demonstrated acceptable fit, *χ*^2^(186) = 1029.59, *p* < 0.001, CFI = 0.870, TLI = 0.853, RMSEA = 0.081 (90% CI [0.076, 0.086]), SRMR = 0.055. The PANAS model showed marginal fit, *χ*^2^(169) = 1284.26, *p* < 0.001, CFI = 0.862, TLI = 0.845, RMSEA = 0.090 (90% CI [0.084, 0.096]), SRMR = 0.075. One item showed a notably weak standardized loading (PANAS.12, *λ* = 0.231), which should be considered in future cross-cultural applications of this instrument. The Flourishing Scale demonstrated good fit, *χ*^2^(20) = 137.33, CFI = 0.946, TLI = 0.925, RMSEA = 0.108 (90% CI [0.091, 0.125]), SRMR = 0.037. The Academic Performance Scale demonstrated excellent fit, *χ*^2^(9) = 40.91, CFI = 0.981, TLI = 0.968, RMSEA = 0.080 (90% CI [0.057, 0.106]), SRMR = 0.025.

The measurement models yielded differential levels of fit across constructs, reflecting both the psychometric characteristics of the instruments and known challenges associated with cross-cultural adaptation.

The social media addiction construct (BSMAS) demonstrated near-acceptable fit, *χ*^2^(9) = 92.13, CFI = 0.925, TLI = 0.876, RMSEA = 0.120, SRMR = 0.042. While the CFI approaches the conventional 0.95 threshold, the TLI falls below 0.90 and the RMSEA exceeds the 0.08 guideline, indicating that fit is adequate but not strong. This supports its use as a reliable latent construct capturing compulsive digital engagement.

The self-image model showed acceptable to marginal fit, consistent with the multidimensional nature of the construct and prior research indicating that self-related constructs often display moderate fit due to their conceptual complexity and hierarchical structure.

Similarly, the PANAS model exhibited marginal fit, which is consistent with previous literature documenting variability in the factor structure of affective measures across cultural contexts and sample characteristics. Despite this, the factor loadings were substantial and theoretically coherent, supporting its inclusion in the structural model.

The authenticity model demonstrated poor fit (CFI = 0.755, TLI = 0.671, RMSEA = 0.170, SRMR = 0.110), which cannot be adequately explained by cross-cultural instability alone. This level of misfit is a meaningful limitation, particularly because authenticity subsequently emerges as the strongest indirect pathway in the structural model. Accordingly, all conclusions regarding authenticity-specific pathways must be interpreted with caution and treated as preliminary. The construct was retained for structural analysis given its theoretical centrality, but this decision should not be taken as an endorsement of the measurement model’s adequacy. Replication with a better-fitting operationalization of authenticity is strongly recommended.

Taken together, the findings suggest that model evaluation should not rely exclusively on global fit indices, but also consider theoretical plausibility, consistency of parameter estimates, and the capacity of the model to capture psychologically meaningful processes in ecologically valid contexts.

### 4.2 Path analysis

A path analysis was conducted using observed composite scores to examine the direct associations between problematic social media use and social media addiction and the outcome variables, as mediated through affectivity, authenticity, and self-image. Rather than a full latent variable structural equation model, the analysis used subscale composites as observed indicators, with self-image represented by its three subscale scores (self-esteem, self-efficacy, and self-achievement). The model was estimated using MLR estimation with Yuan-Bentler robust scaling to account for non-normality, implemented in the lavaan package (Rosseel, 2012). Given that a path model with observed composites was employed rather than a full SEM with latent variables, global model fit indices are not reported for the structural portion of the model; measurement model fit for each individual scale is reported in Table 3.

**Table 3:** Confirmatory factor analysis: robust fit indices for all measurement models (*N* = 940).

Model	*χ*^2^(df)	CFI	TLI	RMSEA	90% CI	SRMR
BSMAS-A—Social Media Addiction (1-factor)	92.13(9)	0.925	0.876	0.120	[0.099, 0.143]	0.042
Authenticity Scale (3-factor)*	928.07(41)	0.755	0.671	0.170	[0.161, 0.180]	0.110
PAS—Self-Image (3-factor)	1029.59(186)	0.870	0.853	0.081	[0.076, 0.086]	0.055
PANAS (2-factor)	1284.26 (169)	0.862	0.845	0.094	[0.090, 0.099]	0.075
Flourishing Scale (1-factor)	137.33 (20)	0.946	0.925	0.108	[0.091, 0.125]	0.037
Academic Performance Scale (1-factor)	40.91 (9)	0.981	0.968	0.080	[0.057, 0.106]	0.025

All indices are based on MLR estimation with Satorra–Bentler scaling. CFI/TLI ≥ 0.95 = excellent, ≥0.90 = acceptable. RMSEA ≤ 0.06 = excellent, ≤0.08 = acceptable. SRMR ≤ 0.08 = excellent, ≤0.10 = acceptable. *Authenticity Scale showed poor fit; observed composite scores were used in the structural model rather than a latent variable. All authenticity-related findings should be interpreted with caution.

Problematic social media use did not significantly predict negative affect (*β* = 0.119, *p* = 0.430), positive affect (*β* = −0.081, *p* = 0.464), or authenticity (*β* = −0.097, *p* = 0.469). In contrast, social media addiction significantly predicted higher negative affect (*β* = 0.265, *p* < 0.001), lower positive affect (*β* = −0.100, *p* = 0.024), and lower authenticity (*β* = −0.373, *p* < 0.001), indicating that compulsive engagement, rather than usage intensity, is associated with affective dysregulation and identity-related vulnerability.

Self-image was not directly predicted by either problematic use (*β* = 0.035, *p* = 0.569) or social media addiction (*β* = −0.013, *p* = 0.741) but was strongly explained by the mediating variables. Negative affect predicted lower self-image (*β* = −0.225, *p* < 0.001), whereas positive affect (*β* = 0.302, *p* < 0.001) and authenticity (*β* = 0.543, *p* < 0.001) predicted higher self-image. Among these, authenticity emerged as the strongest predictor.

Neither problematic use nor social media addiction had significant direct effects on academic performance (*β* = −0.028, *p* = 0.609; *β* = 0.038, *p* = 0.367) or flourishing (*β* = 0.002, *p* = 0.953; *β* = 0.005, *p* = 0.890). In contrast, self-image was a strong positive predictor of both academic performance (*β* = 0.557, *p* < 0.001) and flourishing (*β* = 0.791, *p* < 0.001), supporting a fully mediated pattern of associations.

Taken together, the results support a full mediation pattern in which social media addiction is associated with adaptive outcomes exclusively through affective and identity-related pathways, with no significant direct associations observed for either predictor.

### 4.3 Indirect and total effects

The analysis of indirect effects provided robust support for multiple mediation pathways linking social media addiction to self-image, academic performance, and flourishing. Significant indirect effects from social media addiction to self-image were observed through negative affect (*β* = −0.060, *p* < 0.001), positive affect (*β* = −0.030, *p* = 0.026), and authenticity (*β* = −0.202, *p* < 0.001). Among these, the pathway through authenticity was the strongest.

A complete summary of direct, indirect, and total effects with standard errors, *z*-values, and 95% confidence intervals is presented in Appendix 1. At the level of adaptive outcomes, social media addiction showed significant indirect effects on academic performance through negative affect and self-image (*β* = −0.033, *p* < 0.001), positive affect and self-image (*β* = −0.017, *p* = 0.028), and authenticity and self-image (*β* = −0.113, *p* < 0.001). A similar pattern was observed for flourishing, with significant indirect effects through negative affect (*β* = −0.047, *p* < 0.001), positive affect (*β* = −0.024, *p* = 0.027), and authenticity (*β* = −0.160, *p* < 0.001). Again, the strongest pathway was the one involving authenticity.

By contrast, all indirect effects associated with problematic social media use were non-significant, both for self-image and for the two adaptive outcomes. Total effects further confirmed this distinction: social media addiction showed a significant negative total effect on academic performance (*β* = −0.125, *p* = 0.006) and flourishing (*β* = −0.226, *p* < 0.001), whereas problematic social media use did not show significant total effects.

### 4.4 Hypothesis testing

The results largely support the proposed theoretical model, although not all hypotheses were confirmed in their original direct form.

*H1*, which predicted a direct negative association between social media addiction and self-image, was not supported (*β* = −.013, *p* = .741). No significant direct path was observed between social media addiction and self-image. Significant indirect effects through affectivity and authenticity were identified, indicating that any association between social media addiction and self-image operates entirely through these mediating variables rather than through a direct pathway.

*H2*, stating that affectivity mediates the relationship between social media addiction and self-image, was supported. Both negative affect (*β* = −.060, *p* < .001) and positive affect (*β* = −.030, *p* = .026) significantly mediated this relationship.

*H3*, proposing authenticity as a mediator, was strongly supported. Social media addiction significantly reduced authenticity (*β* = −.373, *p* < .001), and authenticity positively predicted self-image (*β* = .543, *p* < .001). The indirect pathway through authenticity (*β* = −.202, *p* < .001) was the strongest mediation effect in the model.

*H4*, stating that self-image mediates the relationship between social media addiction and academic performance, was supported. Although no direct effect of addiction on academic performance was observed, significant indirect effects through self-image were identified, especially via authenticity (*β* = −.113, *p* < .001).

*H5*, predicting that self-image mediates the relationship between social media addiction and flourishing, was also supported. Significant indirect effects were observed across all pathways, particularly through authenticity (*β* = −.160, *p* < .001).

Overall, the results support a full mediation model in which social media addiction affects academic performance and flourishing indirectly through affective and identity-related mechanisms.

## 5 Discussion

The present study advances a mechanism-based understanding of social media addiction by showing that its cross-sectional associations with academic performance and psychological flourishing are predominantly indirect and consistent with a model in which affective and identity-related processes function as statistical mediators. These findings are based on a cross-sectional design and should not be interpreted as establishing causal statistical mediators or temporal precedence. Rather than supporting exposure-based accounts, the findings align with a growing body of research emphasizing that the psychological impact of digital environments depends less on time spent online and more on how engagement is experienced and internalized (Orben and Przybylski, 2019; Valkenburg, 2022).

A central contribution of the study lies in the clear differentiation between problematic social media use and social media addiction. While previous studies have often treated usage intensity as a primary predictor of outcomes, the present findings show that general use is not significantly associated with affective, identity-related, or adaptive variables. This result is consistent with recent meta-analytic and large-scale studies indicating that simple exposure has weak and inconsistent effects on well-being (Huang, 2017; Orben and Przybylski, 2019). In contrast, social media addiction, conceptualized as compulsive, reward-driven engagement, emerges as a robust predictor of maladaptive outcomes, supporting models of behavioral addiction that emphasize dysregulated reward processing and impaired self-control (Andreassen et al., 2016b; Marino et al., 2018).

The affective pathway represents a first key mechanism through which addiction exerts its influence. The finding that social media addiction was associated with higher negative affect and was associated with lower positive affect is consistent with prior research linking problematic digital engagement to psychological distress and reduced emotional well-being (Kross et al., 2013; Ciacchini et al., 2023). From a theoretical perspective, this pattern aligns with the broaden-and-build framework (Fredrickson, 2001), suggesting that reduced positive affect limits psychological resources, while increased negative affect amplifies vulnerability. Moreover, these findings resonate with process-based models of social media use, which emphasize emotional reactivity and feedback sensitivity as central mechanisms of digital engagement (Valkenburg, 2022).

A second major contribution concerns the role of authenticity as a central identity-related mechanism. The observed negative association between social media addiction and authenticity is consistent with prior work on self-presentation and identity performance in digital environments (Reinecke and Trepte, 2014; Twomey and O’Reilly, 2017). However, the present study extends this literature by demonstrating that authenticity functions as a primary mediator linking addictive engagement to self-image. This finding supports theoretical models of self-discrepancy (Higgins, 1987) and suggests that repeated exposure to evaluative feedback and social comparison may progressively weaken the alignment between internal experience and external expression.

Self-image emerges as the central integrative construct of the model, functioning as a psychological hub through which affective and identity-related processes are translated into adaptive outcomes. This result is consistent with research demonstrating that self-concept clarity and self-evaluation are critical predictors of both academic performance and psychological well-being (Campbell et al., 1996; Diener et al., 2010). Importantly, the present findings extend this literature by situating self-image within a structural mediation framework, showing that it is not directly shaped by digital exposure, but by the emotional and identity-related processing of digital experiences.

The absence of direct effects of social media addiction on academic performance and flourishing is consistent with a full mediation interpretation, though several alternative explanations should be considered. In cross-sectional SEM, the disappearance of a direct path when mediators are included may reflect genuine psychological mediation, but it may also result from multicollinearity among correlated self-report predictors and mediators, or from the specific operationalization of the predictor. Longitudinal designs are required to distinguish between these possibilities. Similarly, the absence of any significant direct effect of H1 (social media addiction ≥ self-image) warrants direct acknowledgment: this may indicate that the addiction-to-self-image relationship is genuinely indirect, but it is also consistent with the possibility that the cross-sectional design and shared method variance have suppressed or redistributed variance across the mediation chain. In this sense, the results reinforce the idea that maladaptive outcomes arise when digital engagement was linked to lower internal regulatory systems rather than through exposure alone.

The analysis of indirect effects provides additional support for a chain mediation mechanism. Social media addiction influences adaptive outcomes through multiple pathways involving affectivity, authenticity, and self-image. Among these, authenticity emerged as the strongest mediator, suggesting that identity-related indirect pathways may play a more central role than purely emotional processes. This finding contributes to an emerging line of research emphasizing the importance of identity coherence and self-concept stability in digital contexts (Reinecke and Trepte, 2014).

The absence of significant effects for the SMU predictor across all pathways in the model merits careful interpretation. While this pattern is consistent with theoretical arguments that general social media use is not inherently harmful (Orben and Przybylski, 2019), it may also reflect measurement limitations of the SMU scale itself. As noted in the Methods section, this scale captures usage intensity and patterns rather than compulsive or addictive engagement in a validated clinical sense, and its construct validity requires further investigation. The contrast between null findings for SMU and significant findings for BSMAS is theoretically interesting, but should not be treated as conclusive evidence distinguishing exposure-based from addiction-based accounts until the SMU measure has been more rigorously validated. Instead, the quality of engagement, particularly its compulsive and dysregulated nature, determines its psychological impact.

From a broader interdisciplinary perspective, the findings are conceptually consistent with concerns raised in neurorights and digital governance scholarship. It must be emphasized that the study does not directly assess cognitive autonomy, mental integrity, or identity continuity, and no neurocognitive variables were measured. The observed associations, between social media addiction and lower authenticity and self-image, are analogous to the kinds of psychological processes that neurorights frameworks are concerned with, but they do not constitute empirical evidence for such constructs or for any form of rights violation. These connections should be read as interpretive and speculative rather than as empirical conclusions. These results suggest that the most significant effects of algorithmically mediated environments may occur at the level of internal psychological processes, which are not fully addressed by current regulatory frameworks focused on data protection and transparency.

Overall, the study contributes to a shift toward mechanism-based models of digital behavior by integrating affective, identity-related, and regulatory processes into a unified explanatory framework. By linking psychological evidence with broader conceptual debates, it provides a more comprehensive understanding of how social media environments shape human functioning in the digital age.

## 6 Limitations

Several limitations should be considered when interpreting the present findings, although they do not diminish the theoretical and empirical contributions of the study. Future research should also incorporate demographic covariates to control for potential confounding effects.

First, the cross-sectional design limits causal inference. Although the structural model is theoretically grounded and consistent with prior literature, the observed relationships remain correlational. It is therefore not possible to determine whether social media addiction was associated with affective dysregulation and identity vulnerability, or whether individuals with pre-existing emotional or identity-related difficulties are more prone to addictive digital engagement. This limitation is particularly relevant from a neurocognitive perspective, as the temporal dynamics between reward sensitivity, emotional reactivity, and regulatory control cannot be directly disentangled. Longitudinal and experimental designs are required to establish temporal precedence and clarify causal mechanisms.

Second, all variables were self-reported by the same participants in a single online survey session, introducing a substantial risk of common method variance (Podsakoff et al., 2003). When all predictors, mediators, and outcomes share the same measurement source, method, and occasion, covariance among variables may be inflated by systematic response tendencies rather than reflecting true construct relationships. This is a particularly serious concern for the mediation pathways reported here, where the entire explanatory model rests on associations among subjective self-report indicators. Future studies should incorporate multi-method designs using behavioral digital trace data, informant reports, or psychophysiological measures to reduce reliance on single-source self-report data. Although all instruments demonstrated satisfactory to excellent internal consistency and are well-established in the literature, self-report data may not fully capture the complexity of digital behavior or underlying neurocognitive processes. Future studies should adopt multi-method approaches integrating behavioral indicators (e.g., digital trace data), informant reports, and psychophysiological measures to enhance construct validity.

Third, the use of an online convenience sample limits generalizability. While the sample was relatively large and heterogeneous in age, voluntary participation and recruitment through social media and email channels introduces a meaningful risk of self-selection bias. Individuals who actively use social media are more likely to have encountered the recruitment materials and chosen to participate, which may result in a sample with systematically higher levels of digital engagement than the broader population. This has two specific implications: first, it may restrict range on the social media addiction and SMU variables, potentially attenuating observed associations; second, it may inflate the prevalence of adverse outcomes associated with high social media use relative to what would be observed in a more representative sample. Replication using probability-based or stratified sampling methods is necessary to establish external validity. Replication across more diverse, representative, and cross-cultural samples is necessary to strengthen external validity.

Fourth, problematic social media use was assessed using a scale developed within the present study. Although the instrument showed adequate internal consistency and conceptual alignment with the theoretical model, further validation is required. Future research should examine its factorial structure, convergent validity, and discriminant validity across independent samples.

Fifth, although the model integrates key affective and identity-related variables, it does not exhaust the range of relevant psychological and neurocognitive mechanisms. Constructs such as impulsivity, executive control, social comparison orientation, and fear of missing out (FoMO) may further refine the explanatory framework. In particular, the absence of direct neurocognitive measures represents a limitation, as processes such as reward sensitivity, attentional bias, and inhibitory control are inferred rather than directly assessed.

The structural model does not include potentially important demographic and contextual covariates. The sample is highly heterogeneous in age (18–72 years), gender, and likely educational status, yet no controls for these variables were included. Age, gender, student status, and platform-specific engagement patterns could plausibly confound both predictor and outcome variables, and their omission represents a simplification that limits the interpretability of the model’s estimates. Future research should incorporate these covariates as standard controls.

Finally, although the structural model demonstrated strong theoretical coherence, no alternative models were formally estimated or compared in the present study. Models such as reciprocal or bidirectional pathways, or a model in which addiction is positioned as an outcome rather than a predictor of affective and identity-related variables, remain untested. The present model should therefore be understood as a theoretically motivated candidate structure rather than as a confirmed account, and future research should compare it against plausible competing specifications using fit-based model selection criteria (e.g., AIC, BIC, or chi-square difference tests).

Additionally, the CFA of the SMU composite scale revealed near-zero and negatively signed factor loadings across platform indicators, suggesting the measure lacks unidimensional construct validity in its current form. This further cautions against strong interpretation of null findings for this predictor.

## 7 Future directions

Building on these limitations, several directions for future research can be identified, with particular relevance for advancing both psychological and neurocognitive understanding of digital behavior.

First, longitudinal designs are essential to clarify the temporal dynamics of the proposed indirect pathways. Future studies should examine whether social media addiction prospectively predicts changes in affectivity, authenticity, and self-image, and whether these changes subsequently influence academic performance and psychological flourishing. Such approaches would enable stronger causal inference and a more precise understanding of developmental trajectories.

Second, experimental and intervention-based research represents a critical next step. Interventions targeting emotional regulation, authenticity, or self-image could be tested to determine whether modifying these variables reduces vulnerability to addictive engagement. From a neurocognitive perspective, interventions targeting executive control, attentional regulation, and reward sensitivity, such as cognitive training or mindfulness-based approaches, may be particularly effective.

Third, future research should integrate objective behavioral measures of digital use. Combining self-report data with digital trace data (e.g., screen time, frequency of checking, interaction patterns) would allow a more precise distinction between usage intensity and addictive engagement, while also improving ecological validity.

Fourth, platform-specific dynamics warrant further investigation. Different platforms operate through distinct affordances and reward structures, which may differentially influence affective and identity-related processes. Future studies should examine whether the statistical mediators identified in the present model vary across platforms such as Instagram, TikTok, or Reddit, particularly in relation to social comparison and algorithmic amplification.

Fifth, extending the model to include additional psychological and personality-related variables would provide a more nuanced understanding of individual differences. Constructs such as self-control, FoMO, narcissism, and social comparison orientation may function as moderators or additional mediators, shaping both vulnerability and resilience.

Sixth, integrating neuroscientific methods represents a particularly promising direction. Techniques such as functional neuroimaging (fMRI), electroencephalography (EEG), and psychophysiological measures could provide direct evidence for the neural correlates of reward processing, emotional reactivity, and self-referential processing in digital contexts, moving beyond theoretical inference toward empirical validation.

Finally, cross-cultural research is essential for assessing generalizability. Cultural norms related to self-presentation, emotional expression, and social comparison may shape both patterns of social media use and their psychological consequences. Comparative studies across cultural contexts would contribute to a more comprehensive and globally relevant understanding of digital behavior.

## 8 Conclusion

The present study demonstrates that the psychological impact of social media is not primarily driven by exposure or time spent online, but by compulsive, addiction-like engagement and the approaches through which this engagement is internalized. Specifically, social media addiction, rather than general use, was associated with increased negative affect, reduced positive affect, and diminished authenticity, which in turn influenced self-image. Self-image emerged as the central mechanism linking these processes to academic performance and psychological flourishing, supporting a full mediation model.

A key contribution of the study lies in identifying authenticity and self-image as core pathways through which digital behavior affects adaptive outcomes. These findings advance a shift from exposure-based explanations toward mechanism-based models that integrate affective regulation and identity processes.

At a broader level, the results provide an empirically grounded perspective relevant to neurorights and digital governance debates. While not constituting direct evidence of rights violations, the identified ways are consistent with concerns related to cognitive autonomy, mental integrity, and identity continuity in algorithmically mediated environments.

Overall, the study highlights that understanding digital harm requires moving beyond what users consume to how digital environments shape emotional processes, identity, and self-perception. This perspective provides a foundation for developing more effective psychological interventions and more nuanced approaches to digital regulation.

## Data Availability

The raw data supporting the conclusions of this article will be made available by the authors, without undue reservation.
