# Supplementary material for: Social media addiction and psychological outcomes: the mediating roles of affect, authenticity, and self-image
Source: Front Psychol. 2026 May 18;17:1837689. doi: 10.3389/fpsyg.2026.1837689 (PMC13223112; doi:10.3389/fpsyg.2026.1837689)
Supplement: Supplementary file 1 [file Supplementary_file_1.docx]

**Appendix 1 (Effects and effects decomposition)**

| Outcome |  | Predictor | Estimator | SE | z | p | 95% CI Lo. | 95% CI Up. | Beta |
| --- | --- | --- | --- | --- | --- | --- | --- | --- | --- |
| Negative affect | <- | Problematic SMU | 16.217 | 20.56 | 0.789 | 0.43 | -24.079 | 56.514 | 0.119 |
| Negative affect | <- | Social Media Addiction | 0.434 | 0.07 | 6.226 | < 0.001 | 0.297 | 0.57 | 0.265 |
| Positive affect | <- | Problematic SMU | -10.878 | 14.85 | -0.733 | 0.464 | -39.983 | 18.227 | -0.081 |
| Positive affect | <- | Social Media Addiction | -0.16 | 0.071 | -2.26 | 0.024 | -0.3 | -0.021 | -0.1 |
| Authenticity | <- | Problematic SMU | -1.076 | 1.487 | -0.723 | 0.469 | -3.991 | 1.839 | -0.097 |
| Authenticity | <- | Social Media Addiction | -0.05 | 0.009 | -5.637 | < 0.001 | -0.067 | -0.032 | -0.373 |
| Self image | <- | Problematic SMU | 2.756 | 4.834 | 0.57 | 0.569 | -6.719 | 12.23 | 0.035 |
| Self image | <- | Social Media Addiction | -0.012 | 0.038 | -0.331 | 0.741 | -0.086 | 0.061 | -0.013 |
| Self image | <- | Negative affect | -0.129 | 0.017 | -7.756 | < 0.001 | -0.161 | -0.096 | -0.225 |
| Self image | <- | Positive affect | 0.175 | 0.018 | 9.656 | < 0.001 | 0.14 | 0.211 | 0.302 |
| Self image | <- | Authenticity | 3.797 | 0.495 | 7.675 | < 0.001 | 2.827 | 4.766 | 0.543 |
| Academical performance | <- | Problematic SMU | -2.26 | 4.424 | -0.511 | 0.609 | -10.931 | 6.41 | -0.028 |
| Academical performance | <- | Social Media Addiction | 0.037 | 0.041 | 0.902 | 0.367 | -0.043 | 0.117 | 0.038 |
| Academical performance | <- | Self image | 0.576 | 0.038 | 15.004 | < 0.001 | 0.501 | 0.652 | 0.557 |
| Fluorishing | <- | Problematic SMU | 0.287 | 4.837 | 0.059 | 0.953 | -9.194 | 9.768 | 0.002 |
| Fluorishing | <- | Social Media Addiction | 0.008 | 0.057 | 0.139 | 0.89 | -0.105 | 0.12 | 0.005 |
| Fluorishing | <- | Self image | 1.416 | 0.067 | 21.135 | < 0.001 | 1.284 | 1.547 | 0.791 |
| Self image | <- | Negative affect <- Problematic SMU | -2.084 | 2.666 | -0.782 | 0.434 | -7.309 | 3.14 | -0.027 |
| Self image | <- | Positive affect <- Problematic SMU | -1.905 | 2.617 | -0.728 | 0.467 | -7.034 | 3.223 | -0.024 |
| Self image | <- | Authenticity <- Problematic SMU | -4.085 | 5.665 | -0.721 | 0.471 | -15.189 | 7.019 | -0.052 |
| Academical performance | <- | Self image <- Negative affect <- Problematic SMU | -1.202 | 1.541 | -0.78 | 0.436 | -4.223 | 1.819 | -0.015 |
| Academical performance | <- | Self image <- Positive affect <- Problematic SMU | -1.098 | 1.515 | -0.725 | 0.469 | -4.068 | 1.872 | -0.014 |
| Academical performance | <- | Self image <- Authenticity <- Problematic SMU | -2.355 | 3.277 | -0.719 | 0.472 | -8.778 | 4.068 | -0.029 |
| Fluorishing | <- | Self image <- Negative affect <- Problematic SMU | -2.951 | 3.769 | -0.783 | 0.434 | -10.338 | 4.436 | -0.021 |
| Fluorishing | <- | Self image <- Positive affect <- Problematic SMU | -2.697 | 3.708 | -0.727 | 0.467 | -9.964 | 4.569 | -0.019 |
| Fluorishing | <- | Self image <- Authenticity <- Problematic SMU | -5.784 | 8.016 | -0.722 | 0.471 | -21.494 | 9.927 | -0.041 |
| Self image | <- | Negative affect <- Social Media Addiction | -0.056 | 0.011 | -4.921 | < 0.001 | -0.078 | -0.034 | -0.06 |
| Self image | <- | Positive affect <- Social Media Addiction | -0.028 | 0.013 | -2.225 | 0.026 | -0.053 | -0.003 | -0.03 |
| Self image | <- | Authenticity <- Social Media Addiction | -0.189 | 0.03 | -6.201 | < 0.001 | -0.248 | -0.129 | -0.202 |
| Academical performance | <- | Self image <- Negative affect <- Social Media Addiction | -0.032 | 0.007 | -4.848 | < 0.001 | -0.045 | -0.019 | -0.033 |
| Academical performance | <- | Self image <- Positive affect <- Social Media Addiction | -0.016 | 0.007 | -2.192 | 0.028 | -0.031 | -0.002 | -0.017 |
| Academical performance | <- | Self image <- Authenticity <- Social Media Addiction | -0.109 | 0.018 | -5.97 | < 0.001 | -0.144 | -0.073 | -0.113 |
| Fluorishing | <- | Self image <- Negative affect <- Social Media Addiction | -0.079 | 0.016 | -4.911 | < 0.001 | -0.11 | -0.047 | -0.047 |
| Fluorishing | <- | Self image <- Positive affect <- Social Media Addiction | -0.04 | 0.018 | -2.219 | 0.027 | -0.075 | -0.005 | -0.024 |
| Fluorishing | <- | Self image <- Authenticity <- Social Media Addiction | -0.267 | 0.044 | -6.051 | < 0.001 | -0.353 | -0.181 | -0.16 |
| Total academical performance | <- | Problematic SMU | -6.915 | 9.398 | -0.736 | 0.462 | -25.335 | 11.504 | -0.086 |
| Total Fluorishing | <- | Problematic SMU | -11.145 | 15.707 | -0.71 | 0.478 | -41.929 | 19.64 | -0.08 |
| Total academical performance | <- | Social Media Addiction | -0.12 | 0.044 | -2.735 | 0.006 | -0.206 | -0.034 | -0.125 |
| Total Fluorishing | <- | Social Media Addiction | -0.378 | 0.076 | -4.973 | < 0.001 | -0.527 | -0.229 | -0.226 |
